# GERIATRIC ASSESSMENT ADOPTION IN COMMUNITY CANCER CENTERS: TRENDS, BARRIERS, AND RECOMMENDATIONS

**DOI:** 10.1093/geroni/igz038.446

**Published:** 2019-11-08

**Authors:** Elana Plotkin, Lorna Lucas, Peggy Sue Burhenn, Ginah Nightingale, Kah Poh Loh, Priscilla D Allen, Efrat Dotan

**Affiliations:** 1 Association of Community Cancer Centers, Rockville, Maryland, United States; 2 City of Hope, Duarte, California, United States; 3 Jefferson Health, Philadelphia, Pennsylvania, United States; 4 University of Rochester, Rochester, New York, United States; 5 Louisiana State University, Baton Rouge, Louisiana, United States; 6 Fox Chase Cancer Center, Philadelphia, Pennsylvania, United States

## Abstract

Addressing the needs of older adults with cancer is critical for the delivery of high-quality, patient-centered care. The Association of Community Cancer Centers (ACCC) has identified barriers and best practices for serving this growing patient population in order to help support the multidisciplinary team in understanding and proactively preparing for this large subgroup of patients. A survey was administered to 332 cancer professionals. 95% agreed that their older adult patients would benefit from a comprehensive geriatric assessment, yet only 17% of respondents are performing CGAs. 74% of respondents are not using any screening tool to identify high risk patients. The top three barriers to this were time/personnel and limited familiarity with available, validated tools. 61% are not focused on increasing older adult participation in clinical trials which leads to a disparity in care. Techniques for evaluating fitness, cognitive status, psychological status, comorbidities, and toxicity risk were often informal and not recorded in an EMR. Three in-depth focus groups were completed at programs demonstrating effective, yet different models of care for an older population. City of Hope Cancer Center is running a Senior Adult program under a grant where patients receive care in concordance with a score (CARG toxicity calculator) and a team review with a geriatrician. Sidney Kimmel Cancer Center has a consultative clinic where patients attend a 2-hour appointment to complete a comprehensive geriatric assessment with oncology, geriatrics, and specialists including pharmacy and nutrition. ACCC has recommended resources to address deficits in care, particularly in the community setting.

